# Analysis of prevalence of adverse events connected with anti-tuberculosis drugs during pregnancy: A meta-analysis

**DOI:** 10.1016/j.heliyon.2023.e22786

**Published:** 2023-11-23

**Authors:** Diqing Wu, Xiaobei Li, Hui Wan, Ashwag Shami, Hassan H. Alhassan, Maher M. Al-Enazi, Shehla Nasar Mir Najib Ullah, Abdulqadir J. Nashwan, Shahanavaj Khan

**Affiliations:** a Department of Obstetrics, Nanjing Drum Tower Hospital the Affiliated Hospital of Nanjing University Medical School, Nanjing, 210008, China; b Department of Biology, College of Science, Princess Nourah Bint Abdulrahman University, Riyadh, 11671, Saudi Arabia; c Department of Clinical Laboratory Science, College of Applied Medical Sciences, Jouf University, Sakaka, Saudi Arabia; d Department of Medical Laboratory Science, College of Applied Medical Sciences, Prince Sattam Bin Abdulaziz University, Al-Kharj, 11942, Saudi Arabia; e Department of Pharmacognosy, Faculty of Pharmacy, King Khalid University, Abha, Saudi Arabia; f Hamad Medical Corporation, Doha, Qatar; g Department of Medical Lab Technology, Indian Institute of Health and Technology (IIHT), Deoband, 247554, Saharanpur, UP, India; h Department of Health Sciences, Novel Global Community Educational Foundation, NSW, Australia

**Keywords:** Tuberculosis, Safety, Anti-tuberculous drugs, Pregnancy, Adverse events

## Abstract

**Background:**

*Mycobacterium tuberculosis* infection is transmitted among humans via airborne droplets. The drugs used in the initial treatment regimen for tuberculosis (TB) cross the placenta, raising some concerns regarding their safety during pregnancy may provide a more valid approach for evaluating the relative influence of various risk factors. Adverse events of anti-tuberculous (anti-TB) drug during pregnancy remain uncertain and controversial issues.

**Methods:**

We performed a systematic analysis to study the adverse events connected with anti-TB drugs usage during pregnancy. The risk of bias in the included studies was assessed using the Cochrane Collaboration criteria. Interstudy heterogeneity was assessed via Cochran's test. Assuming heterogeneity, a random-effects model was applied. Outcomes were pooled using the inverse variance method. Besides, a funnel plot was created to assess publication bias. We used Egger's linear regression test of funnel plot asymmetry, modified to accommodate inter-study heterogeneity. Effect estimates and confidence intervals for all studies were depicted on a forest plot.

**Results:**

The prevalence of total adverse events for all anti-TB drugs was 25.9 %. According to the drug category, the prevalence of total adverse events was 50 % for ethambutol, 32.6 % for the six-month directly observed treatment short-course (DOTS), 31.4 % for the nine-month DOTS, and 13.7 % for isoniazid.

**Conclusions:**

There is a high rate of reported adverse events associated with anti-TB drugs usage during pregnancy. We concluded that more high-quality clinical studies and research works are needed to reach a conclusive decision on the safety of the treatment of TB among pregnant women.

## 1 Introduction

Among infectious diseases, tuberculosis (TB) is the leading cause of death globally. About 1.7 million TB-infected patients died in 2004 [1,2]. In 2015, about 10.4 million new cases were reported, among which 56 % were men, 34 % were women, and 10 % were children [3]. The highest incidence rates are observed in the continent of Africa (∼365 new cases per 100,000 populations per year). Also, highly populous Asian countries like China, Bangladesh, India, Indonesia, and Pakistan are responsible for about half (48 %) of the new cases every year. In countries with high incidence rates of TB, most patients are young adults, while in countries with low incidence rates, the older population is generally affected due to reinfection or latent infection. It was estimated that from 2004 onward, the global incidence rates of TB will increase continuously, especially in Africa [4].

Tuberculosis is primarily transmitted through the inhalation of airborne droplets containing Mycobacterium tuberculosis [5]. The risk factors for TB transmission and infection include close contact with individuals who have active TB, compromised immune systems, and conditions such as malnutrition and HIV/AIDS [6]. Recent epidemiological data have emphasized the significance of drug-resistant strains of TB, making its management and treatment during pregnancy an even more complex challenge [7]. As a challenging infectious disease, TB is considered an important health issue during pregnancy. The prevalence of TB amongst pregnant women is estimated from 0.26 % to 7.2 %; it is more prevalent (11 %) among HIV-infected women [8]. The problem is more significant in developing countries [9]. South Asia and sub-Saharan Africa are the high incident regions, though between 2003 and 2011, the TB incidence rate among women in the United States rose from 1.92 to 4.06 per 10,000 births [10]. In low- and middle-income countries, HIV/AIDS, maternal conditions, and TB are responsible for about 50 % of death in women in their reproductive ages [11]. Furthermore, about 28 % of maternal deaths are due to non-obstetrical causes like infectious diseases [12]. It is estimated that about 216,500 pregnant women had TB globally in 2011. There is no data concerning the number of pregnant women afflicted by multidrug-resistant TB (MDR-TB) [8].

The drugs used in the initial treatment regimen for TB cross the placenta, raising some concerns regarding their safety during pregnancy [13].Despite the probable high rate of prevalence of adverse events of anti-TB drugs in pregnancy, it seems that postponing treatment leads to a relatively higher rate of complications [14]. It is known that untreated TB increases the risk of low birth weight and that the infant of pregnant mothers may be born with the disease [15].

There are inadequate documents concerning the safety, applicability, and outcomes of medical treatments in pregnant women with TB [16].In fact, we do not have enough evidence to evaluate the teratogenicity of anti-TB drugs [17].Furthermore, guidelines for MDR-TB are based only on few case reports and case series and no guidelines exist regarding the management of MDR-TB in pregnancy [16,18,19]. More research is needed to evaluate the adverse events associated with the treatment of TB and MDR-TB in high-risk populations during pregnancy [20]. In this study, we performed a systematic review and meta-analysis of the adverse events associated with anti-TB drug usage during pregnancy.

## 2 Methods

### 2.1 Data sources and search strategy

Human studies reporting the use of anti-TB drugs in pregnant women were sought in the PubMed, Scopus, Embase, and Cochrane databases. The search strategy included the terms “pregnancy or pregnant” and “tuberculosis or TB” or “isoniazid, rifampin, ethambutol, or pyrazinamide", looked up in the subject, abstract, and keywords. Studies published from the inception of the databases to December 30, 2022, were included in the current study. The literature search was limited to articles in English. The titles and abstracts of all retrieved papers were screened regarding their relevance to the topic, with the full text of relevant manuscripts being subsequently assessed for eligibility. Articles that were not available online in the form of full text were requested from the corresponding author via email.

### 2.2 Eligibility criteria

Human studies reporting the use of anti-TB drugs by pregnant women with or without comorbidities (e.g., HIV) were included. Animal studies, reviews, letters, commentaries, case studies, and clinical cohorts without appropriate controls were excluded. In the case of duplicated reports, the ones with more presented data were used in the data extraction.

### 2.3 Data extraction

The screening of full-text papers for data extraction was carried out independently by two reviewers based on a predefined protocol including bibliographic information, location and design of the study, mean age of the enrolled participants, the number of enrolled participants in each group, the intervention, and the comparison.

### 2.4 Risk of bias in individual studies

Two reviewers independently assessed the risk of bias among the included studies. We assessed the risk of bias of the included studies using Cochrane Collaboration criteria. Detection bias, performance bias, attrition bias, selection bias (random sequence generation and allocation concealment), and reporting bias were assessed along with other forms of bias.

### 2.5 Statistical analysis

Inter-study heterogeneity was assessed using Cochran's test with a significance level of less than 0.1. Assuming heterogeneity, a random-effects model was applied. Outcomes were pooled using the inverse variance method. Besides, a funnel plot was created to assess publication bias. We also made use of Egger's linear regression test of funnel plot asymmetry, modified to accommodate inter-study heterogeneity. Effect estimates and confidence intervals (0.95 CI) for all studies were depicted on a forest plot. All analyses were performed using version 2.0 of the Comprehensive Meta-Analysis (CMA) software [21].

## 3 Results

### 3.1 Description of search

**Fig. 1:**
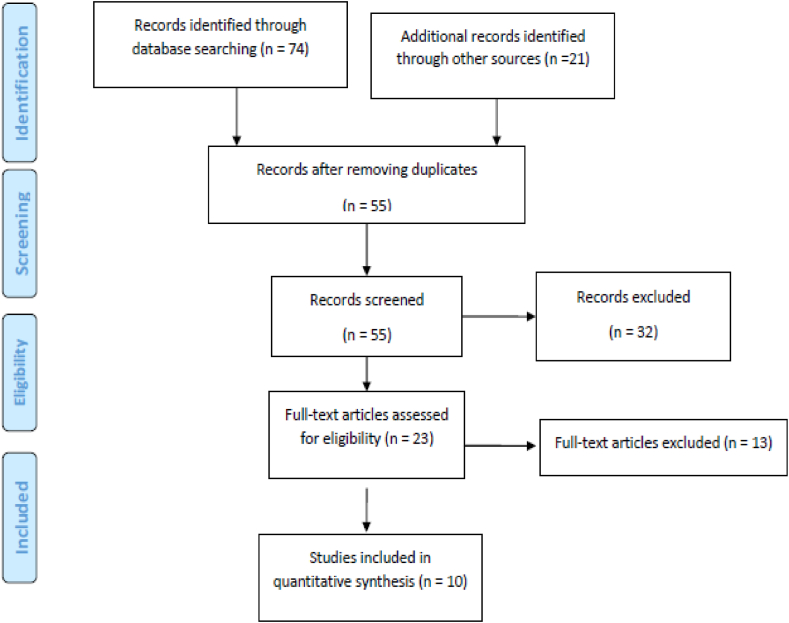
PRISMA flow diagram for the investigated studies.

We identified a total of 95 records after searching the databases. After the removal of 40 duplicates, the title and abstracts of 55 records were screened. Subsequently, 32 papers were excluded and 23 were prepared for full-text screening. A total of 13 records were excluded based on eligibility criteria in this stage. Finally, 10 studies were included in our current analysis (Fig. 1).

### 3.2 Characteristics of the included studies

According to the geographical area, three studies were conducted in the USA, three in South Africa, one in Hungary, one in Mexico, one in India, and one was multinational (USA, Canada, Brazil, Spain, and Peru). The basic characteristics included in the studies are summarized in Table 1.

**Table 1:** Basic characteristics of the included studies.

Article	Country	Sample size	Study design	Population	Intervention	Comparison
(Chang et al., 2013)	USA	3918	Retrospective study	Latent tuberculosis infection patients	Isoniazid 300 mg tablets daily	Home follow-up vs. clinical follow-up
(Gupta et al., 2019)	USA	956	Multicenter, double-blinded, and placebo-controlled	Pregnant women with HIV infection	Isoniazid preventive therapy	28 weeks, initiated either during pregnancy (immediate group) vs. at week 12 after delivery (deferred group).
(Kalk et al., 2018)	South Africa	7310	Cohort	Pregnant women living with HIV	Isoniazid preventive therapy	Isoniazid preventive therapy vs. no isoniazid preventive therapy
(Moro et al., 2018)	USA, Canada, Brazil, Spain, and Peru	87	Phase 3, open-label, randomized trial that compared 3HP to 9H for treatment of LTBI	Pregnant women	12-dose, once-weekly isoniazid (H, 900 mg) plus rifapentine (P, 900 mg) (3HP)	3HP vs. 9 months of daily isoniazid (H, 300 mg) (9H)
(Salazar-Austin et al., 2020)	South Africa	155	Prospective observational cohort	Pregnant women living with HIV	Isoniazid preventive therapy	Isoniazid preventive therapy vs. no isoniazid preventive therapy
(Taylor et al., 2013)	USA	196	Nested study in a placebo-controlled trial	HIV-infected women	36 months daily isoniazid	Isoniazid vs. no isoniazid
(Czeizel A et al., 2001)	Hungary	10	National-based registry of congenital abnormalities	Pregnant women	Isoniazid	Isoniazid vs. ethambutol
(Figueroa-Damian and Arredondo-Garcia 1998)	Mexico	100	Cohort	Pregnant women	TB drugs	Early treatment vs. late treatment among pregnant women afflicted with TB
(Sharma et al., 2016)	India	175	Randomized controlled trial	Pregnant women	DOTS	DOTS 6 months vs. DOTS 9 months
(Loveday et al., 2020)	South Africa	108	Observational cohort study	Pregnant women	Multidrug/rifampicin-resistant (MDR/RR)-TB	Bedaquiline exposure vs. no bedaquiline exposure

### 3.3 Quality of the included studies

The risk of bias among the included studies based on Cochrane Collaboration is summarized in Table 2. The overall quality of the included studies was low to moderate.

**Table 2:** The risk of bias of the included studies using Cochrane Collaboration criteria for quality assessment.

Item/Author, year	*Chang, 2013*	Gupta, 2019	Kalk, 2020	Moro, 2018	Salazar-Austin, 2019	Taylor, 2013	Figueroa-Damia, 1998	Sharma, 2016	Loveday, 2020	Czeizel, 2001
Random sequence generation (selection bias)	**H**	**L**	**H**	**L**	**H**	**L**	**H**	**L**	**H**	**H**
Allocation concealment (selection bias)	**U**	**L**	**H**	**L**	**H**	**L**	**H**	**L**	**H**	**H**
Blinding of participants and personnel (performance bias)	**H**	**L**	**H**	**L**	**H**	**H**	**H**	**L**	**U**	**L**
Blinding of outcome assessment (detection bias)	**H**	**L**	**U**	**U**	**U**	**H**	**L**	**L**	**H**	**H**
Incomplete outcome data (attrition bias)	**L**	**L**	**L**	**L**	**H**	**L**	**L**	**L**	**L**	**L**
Selective reporting (reporting bias)	**H**	**U**	**L**	**H**	**H**	**U**	**U**	**L**	**U**	**U**
Other bias	**H**	**L**	**H**	**L**	**U**	**L**	**H**	**U**	**H**	**H**

H: high; L: low; U: unclear.

### 3.4 The results of the meta-analysis

#### 3.4.1 Total adverse events

**Fig. 2:**
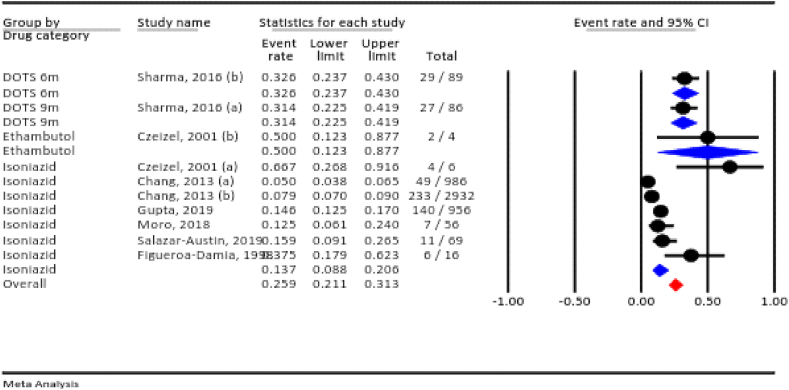
The total adverse events rate for all anti-TB drugs during pregnancy.

Among the included studies, seven assessed total adverse events. The prevalence of total adverse events for all TB drugs during pregnancy was 25.9 % (95 % CI: 21.1–31.3 %, I^2^ = 94.6 %, *P* < 0.001). According to the drug category, the prevalence of total adverse events was 50 % for ethambutol, 32.6 % for the six-month directly observed treatment short-course (DOTS), 31.4 % for the nine-month DOTS, and 13.7 % for isoniazid (Fig. 2).

#### 3.4.2 Neonatal death

**Fig. 3:**
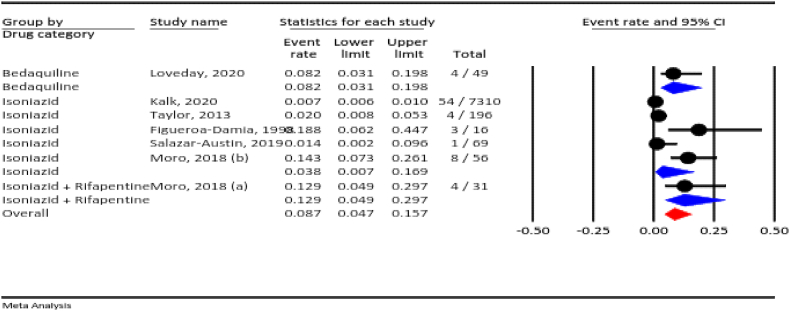
The rate of neonatal death for all anti-TB drugs during pregnancy.

The results of the meta-analysis showed that the prevalence of neonatal death among all pregnancies in which anti-TB drugs were used was 8.7 % (95 % CI: 4.7–15.7 %, I^2^ = 94.7 %, *P* < 0.001). According to the drug category, the prevalence of neonatal death was 3.8 % for isoniazid, 12.9 % for isoniazid plus rifapentine, and 8.2 % for bedaquiline (Fig. 3).

#### 3.4.3 Abortion

**Fig. 4:**
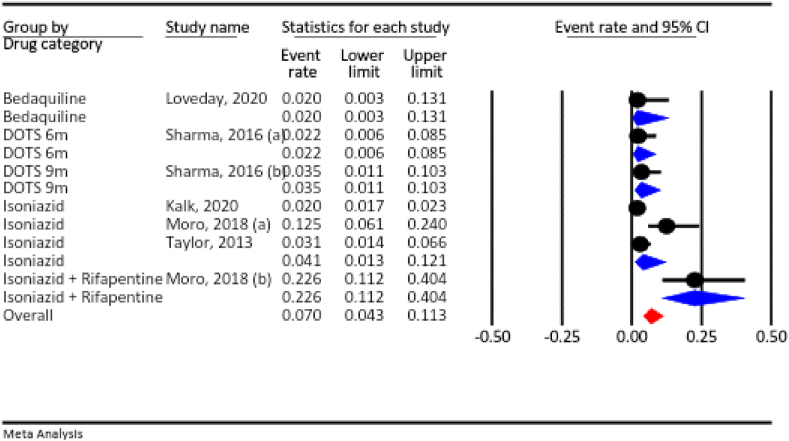
The rate of abortion for all anti-TB drugs in pregnancy.

Based on our meta-analysis, the prevalence of abortion across all pregnant women who used anti-TB drugs was 7.0 % (95 % CI: 4.3–11.3 %, I^2^ = 89.7 %, *P* < 0.001).According to the drug category, the prevalence of abortion was 4.1 % for isoniazid, 22.6 % for isoniazid plus rifapentine, 2.0 % for bedaquiline, 2.2 % for the six-month DOTS, and 3.5 % for the nine-month DOTS (Fig. 4).

#### 3.4.4 Congenital anomalies

**Fig. 5:**
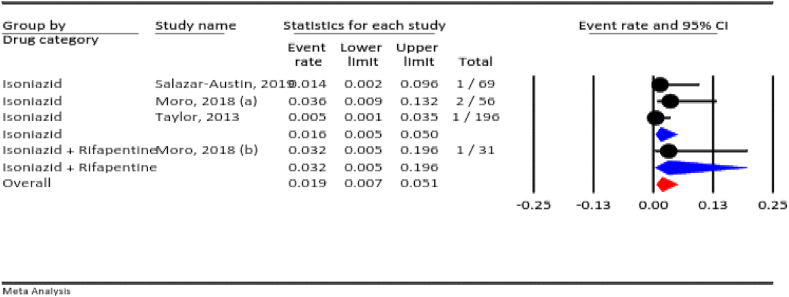
Rate of congenital anomalies for all anti-TB drugs in pregnancy.

The results of our meta-analysis revealed that the prevalence of congenital anomalies in all anti-TB drugs used during pregnancy was 1.9 % (95 % CI: 0.07–5.1 %, I^2^ = 0.0 %, *P* = 0.404). According to the drug category, the prevalence of congenital anomalies was 1.6 % forisoniazid and 3.2 % for isoniazid plus rifapentine (Fig. 5).

#### 3.4.5 Drug-induced hepatitis

**Fig. 6:**
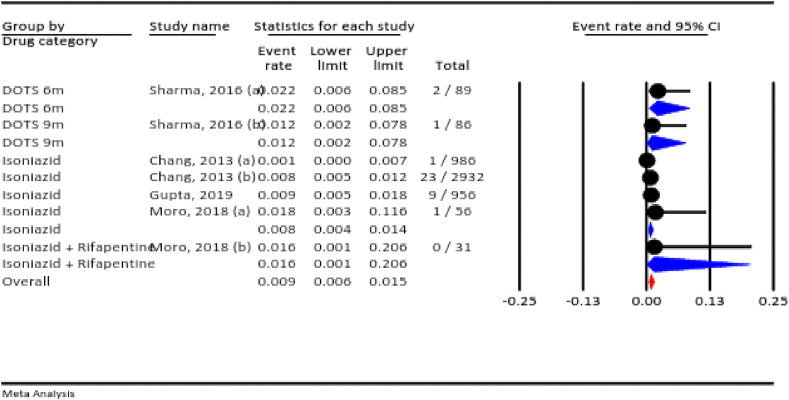
The rate of drug-induced hepatitis for all anti-TB drugs in pregnancy.

Based on our meta-analysis, the overall prevalence of hepatitis in pregnant women using anti-TB drugs was 0.9 % (95 % CI: 0.6–1.5 %, I^2^ = 20.3 %, *P* = 0.274). According to the drug category, the prevalence of hepatitis was 0.8 % for isoniazid, 1.6 % for isoniazid plus rifapentine, 2.2 % for the six-month DOTS, and 1.2 % for the nine-month DOTS (Fig. 6).

#### 3.4.6 Low birth weight (LBW)

**Fig. 7:**
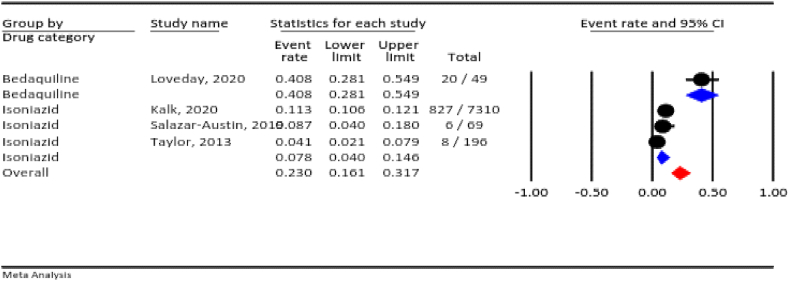
The rate of low birth weight for all anti-TB drugs in pregnancy.

According to our meta-analysis, the prevalence of LBW across all anti-TB drugs used during pregnancy was 23.0 % (95 % CI: 16.1–31.7 %, I^2^ = 93.1 %, *P* < 0.001). In terms of the drug category, bedaquiline was responsible for most cases of LBW (40.8 %), after which cameisoniazid (7.8 %) (Fig. 7).

#### 3.4.7 Stillbirth

**Fig. 8:**
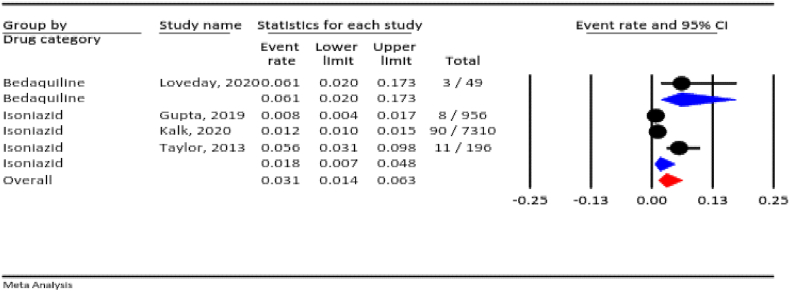
The rate of stillbirth for all anti-TB drugs in pregnancy.

Based on meta-analysis, the prevalence of stillbirth in all TB drugs during pregnancy was 3.1 % (95 % CI: 1.4–6.3 %, I^2^ = 90.4 %, *P* < 0.001). According to the drug category, the prevalence of stillbirth was 1.8 % for isoniazid and 6.1 % for bedaquiline (Fig. 8).

#### 3.4.8 Preterm delivery

**Fig. 9:**
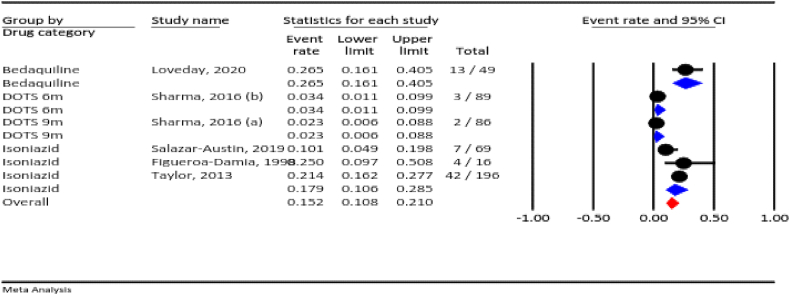
The rate of preterm delivery for all anti-TB drugs in pregnancy.

Our meta-analysis revealed that the prevalence of preterm delivery in all TB drugs used during pregnancy was 15.2 % (95 % CI: 10.8–21.0 %, I^2^ = 81.2 %, *P* < 0.001). Categorically, bedaquiline was responsible for the highest rate of preterm delivery (26.5 %), followed by isoniazid/rifapentine (22.6 %), isoniazid (17.9 %), six-month DOTS (3.4 %), and nine-month DOTS (2.3 %) (Fig. 9).

#### 3.4.9 Elevated liver enzymes

**Fig. 10:**
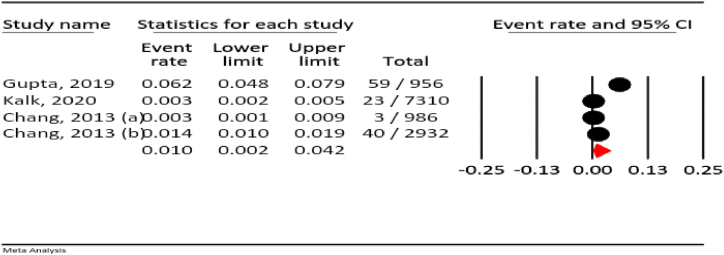
The rate of elevated liver enzymes for all anti-TB drugs in pregnancy.

The results of our meta-analysis show that the prevalence of elevated liver enzymes in isoniazid use during pregnancy was 1.0 % (95 % CI: 0.2–4.2 %, I^2^ = 98.2 %, *P* < 0.001) (Fig. 10).

#### 3.4.10 Complications of isoniazid vs. control

**Fig. 11:**
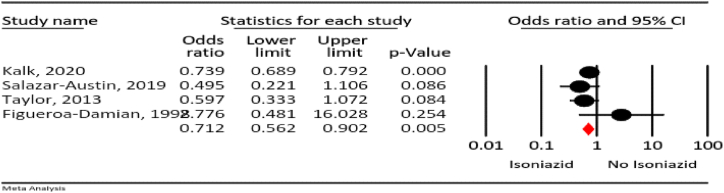
The odds ratio for the risk of total adverse events for isoniazid in pregnancy compared to other therapeutic regimens.

Our meta-analysis revealed that the rate of total adverse events was 29 % lower in the isoniazid group than the control group (OR = 0.71, 95 % CI: 0.56–0.90; *P* = 0.005, I^2^ = 17.6 %, *P* = 0.303) (Fig. 11).

#### 3.4.11 Publication bias

Publication bias was not observed among the included studies according to the results of both the Egger test (P = 0.173) and Begg's test (P = 0.721).

## 4 Discussion

Active TB is the prime cause of maternal mortality throughout the world [3]. Pregnancy can be considered a risk factor for the disease as a study in the United Kingdom showed higher incidence rates of TB infection in pregnant women relative to their non-pregnant counterparts [22]. To increase tolerance to fetal antigens, a kind of immunosuppression is formed during pregnancy via the downregulation of T-helper type 1 (Th1), interferon (IFN)-γ and tumor necrotizing factor (TNF)-α. These changes make the body susceptible to new infections or reactivation of intracellular microbes like *Mycobacterium tuberculosis* [23,24].

During pregnancy, TB is almost mild and subclinical with no or non-specific symptoms; after delivery, new and more severe clinical manifestations present, which is called immune reconstitution syndrome (IRS) [25,26].

Due to the mild symptoms of TB during pregnancy and its similarity to pregnancy symptoms like fatigue and shortness of breath, the diagnosis of the disease is challenging and is mostly confirmed after delivery [[27], [28], [29]]. On the other hand, late diagnosis of TB and late or insufficient treatment during pregnancy can cause mortality and morbidity for both the mother and fetus, signaled by a two-fold increase in premature births and a six-fold increase in perinatal deaths [30,31]. Pregnancy complications like sepsis, severe preeclampsia, eclampsia, placenta previa, postpartum hemorrhage, and anemia are documented 80 % more among TB-infected pregnant women than their non-infected counterparts [32], while hospital admission and miscarriage are nine times more prevalent in the former group relative to the latter [33]. Besides, the risk of TB transmission is high within the first21 days of infant life [34]. Infants of TB-infected women are at high risk of low birth weight, prematurity, and being small for gestational age [31,35,36].

Both the mother and child benefit from early diagnosis and suitable treatment of TB, especially before delivery [37]. Before starting treatment with anti-TB drugs, a risk evaluation of the drug's adverse effects and teratogenicity, the severity of the disease, and the gestational age must be done. The greatest rate of teratogenicity is during the first trimester, and any delays in treatment should be paired with close follow-ups and observations [20].

First-line TB drugs including isoniazid, rifampicin, pyrazinamide, and ethambutol are considered safe during pregnancy and are recommended by the World Health Organization (WHO) [13,38].In contrast, pyrazinamide is not recommended by the Centers for Disease Control and Prevention (CDC) due to inadequate data about its safety during pregnancy [39]. Evidence about the safety of first-line drugs is limited, so more investigations should be done [18,19]. Until now, no evidence is available about rifamycin-based regimens during pregnancy [40]. Second-line drugs can be used during pregnancy but little evidence is available regarding their safety in the mentioned period [16,20]. The CDC recommends liver function test assessment before the initiation of anti-TB treatment and monthly thereafter [41]. Due to the risk for ototoxicity and fetal malformations, aminoglycosides are FDA class D and should be avoided during pregnancy, especially in the first 20 weeks. Ethionamide and prothionamide can increase the risk of nausea and vomiting during pregnancy [20]. Other second-line drugs including amoxicillin/clavulanate, meropenem, and bedaquiline are considered as class B(no human studies and no risks reported in animal studies) and class C(no human studies but risks reported in animal studies) [20].

A previous report by Hamada et al. evaluated the safety of isoniazid preventive treatment in pregnant and postpartum women through a meta-analysis. In a total of nine studies, they found pregnant women receiving isoniazid to be at an elevated risk of hepatotoxicity. However, the results were reported to be inconsistent and heterogeneous. Our meta-analysis showed that the rate of total adverse events was 29 % lower in the isoniazid group than in the control group receiving other types of treatment.

The key strengths of our systematic review and meta-analysis were the application of a systematic search with well-defined eligibility criteria, systematic data extraction, and thorough analysis. This study was the first meta-analysis accumulating the available evidence on the safety of all anti-TB drugs for pregnant women. One previous meta-analysis evaluated the safety of only isoniazid in the treatment of these patients. Besides the mentioned strengths, this meta-analysis suffered multiple limitations. The most important limitation of the study was the lack of enough high-quality studies comparing each anti-TB drug against a placebo or the lack of intervention. Another limitation was that a high number of studies were conducted on pregnant women infected by HIV and receiving other medications that may complicate the incidence of adverse events, meaning that conclusions should be drawn with caution.

In this study, given the inadequate evidence regarding the safety of medical treatment in pregnant women with TB, we performed a systematic review and meta-analysis of the adverse events associated with anti-TB drug usage during pregnancy. The prevalence of total adverse events for all anti-TB drugs was 25.9 %. According to the drug category, the prevalence of total adverse events was 50 % for ethambutol, 32.6 % for the six-month DOTS, 31.4 % for the nine-month DOTS, and13.7 % for isoniazid. However, despite the high rate of reported adverse events observed, untreated TB may cause a greater hazard to a pregnant woman and her fetus than its medications. Hence, more high-quality clinical studies are needed to reach a conclusive decision on the safety of treatment of TB during pregnancy.

## Data availability

No data was used for the research described in the article.

## Ethical approval

No ethical clearance was needed for this publication because all information and data were published previously and were anonymized.

## CRediT authorship contribution statement

**Diqing Wu:** Writing – review & editing, Writing – original draft, Methodology, Formal analysis, Data curation, Conceptualization. **Xiaobei Li:** Writing – review & editing, Writing – original draft, Methodology, Formal analysis, Nitesh Kumar Poddar, Writing – review & editing, Writing – original draft, Data curation. **Hui Wan:** Writing – review & editing, Writing – original draft, Methodology, Formal analysis. **Ashwag Shami:** Writing – review & editing, Writing – original draft, Data curation. **Hassan H. Alhassan:** Writing – review & editing, Writing – original draft, Data curation. **Maher M. Al-Enazi:** Writing – review & editing, Writing – original draft, Data curation. **Abdulqadir J. Nashwan:** Writing – review & editing, Writing – original draft. **Shahanavaj Khan:** Writing – review & editing, Writing – original draft, Supervision.

## Declaration of competing interest

The authors declare that they have no known competing financial interests or personal relationships that could have appeared to influence the work reported in this paper.
